# Adverse Events Related to Emergency Department Care: A Systematic Review

**DOI:** 10.1371/journal.pone.0074214

**Published:** 2013-09-12

**Authors:** Antonia S. Stang, Aireen S. Wingert, Lisa Hartling, Amy C. Plint

**Affiliations:** 1 Department of Pediatrics and Community Health Sciences, University of Calgary, Calgary, Canada; 2 Cochrane Child Health Field, Department of Pediatrics, University of Alberta, Edmonton, Canada; 4 Department of Pediatrics and Emergency Medicine, University of Ottawa, Ottawa, Canada; 3 Alberta Research Center for Health Evidence, Department of Pediatrics, University of Alberta, Edmonton, Canada; Iran University of Medical Sciences, Islamic Republic of Iran

## Abstract

**Objective:**

To systematically review the literature regarding the prevalence, preventability, severity and types of adverse events (AE) in the Emergency Department (ED).

**Methods:**

We systematically searched major bibliographic databases, relevant journals and conference proceedings, and completed reference reviews of primary articles. Observational studies (cohort and case-control), quasi-experimental (e.g. before/after) studies and randomized controlled trials, were considered for inclusion if they examined a broad demographic group reflecting a significant proportion of ED patients and described the proportion of AE. Studies conducted outside of the ED setting, those examining only a subpopulation of patients (e.g. a specific entrance complaint or receiving a specific intervention), or examining only adverse drug events, were excluded. Two independent reviewers assessed study eligibility, completed data extraction, and assessed study quality with the Newcastle Ottawa Scale.

**Results:**

Our search identified 11,624 citations. Ten articles, representing eight observational studies, were included. Methodological quality was low to moderate with weaknesses in study group comparability, follow-up, and outcome ascertainment and reporting. There was substantial variation in the proportion of patients with AE related to ED care, ranging from 0.16% (n = 9308) to 6.0% (n = 399). Similarly, the reported preventability of AE ranged from 36% (n = 250) to 71% (n = 24). The most common types of events were related to management (3 studies), diagnosis (2 studies) and medication (2 studies).

**Conclusions:**

The variability in findings and lack of high quality studies on AE in the high risk ED setting highlights the need for research in this area. Further studies with rigorous, standardized outcome assessment and reporting are required.

## Introduction

Patient safety, defined as “freedom from accidental injury”, [1] has been identified as an international priority [1], [2]. Adverse events (AE), defined here as unintended harms resulting from the care and services provided to the patient [3], represent a significant threat to patient safety and public health. Studies conducted in multiple countries have reported a prevalence of AE among hospitalized patients ranging from 2.9% to 16.6%, with 36.9% to 51% of events considered preventable [4], [5], [6], [7], [8].

The emergency department (ED) is considered particularly high risk for AE. Reasons include high patient volume, patient acuity and complexity, a work environment characterized by time constraints, multiple interruptions and disrupted sleep cycles for health care workers, as well as factors such as high risk diagnostic and therapeutic interventions and variable levels of physician training [8], [9], [10], [11]. While population based estimates from the seminal Harvard Medical Practice Study (HMPS) suggest that about 3% of AE occur in the emergency department (ED), this is likely an underestimation given that the study included only hospitalized patients [8] and only 9.5% of patients who visit an ED are admitted [12]. Research on AE in multiple care settings, has identified differences in the prevalence, types, severity and preventability of AE [4], [13], [14], [15], [16], [17]. This discrepancy in findings highlights the importance of setting specific research on AE. As a result of concerns that the ED is a high risk environment for patient safety and that setting specific research is important, we conducted a systematic review to summarize the best available evidence regarding the prevalence, preventability, severity and types of AE related to ED care.

## Methods

The systematic review followed a prospective protocol that was developed a priori.

### Literature Search

The main search strategy (Table S1) was developed by a medical research librarian (AM) in consultation with the research team and content experts. We conducted a systematic search of the literature from 1985 to September 2012. We chose 1985 for the start of our search because we wanted to ensure that health care provided was comparable with care provided at present and because the landmark Harvard Medical Practice studies [7], [8], which focused interest on patient safety and provided a more standardized, rigorous methodology for AE measurement and reporting, were published in 1991. We searched the following bibliographic databases: Medline, Cochrane Library, International Pharmaceutical Abstracts, EMBASE, PubMed, CINAHL and Web of Knowledge. Search terms included those corresponding to patient safety, medical errors and adverse events combined with terms describing the emergency department setting. Other search strategies included searches of relevant conference proceedings and journals (Table S2) for the years 2009–2012, contact with experts in the field, and review of reference lists of included articles and relevant reviews. There was no restriction on publication status but due to resource constraints only those published in English were considered.

### Study Selection

After removal of duplicates (AM) and clearly irrelevant citations (e.g. erectile dysfunction, cancer), two authors (AS and AP) independently reviewed the titles and abstracts generated by the search to identify potentially relevant articles. The same two reviewers then independently assessed the full article for inclusion using pre-defined eligibility criteria. Eligibility criteria included study design, AE definition, AE measurement, study population and study setting. For study design we considered observational (cohort or case-control), quasi-experimental or randomized controlled trials (RCTs). Recognizing that RCTs by definition include a highly selected patient population, we decided a priori that we would only include RCTs in which the control group(s) provided data on the proportion of AE occurring in patients representative of the general ED population. For example, we would include data from the control group of a cluster randomized controlled trial with EDs/hospitals randomized to the intervention (an intervention designed to reduce AE) or control (usual care) group. Included studies also had to have a clearly specified definition of AE reflecting unintended harms resulting from the care and services provided to the patient [3] [7], [8]. Included studies also had to address both the measurement of adverse events and the measurement of the association of these events with the ED care provided. With respect to study population, included studies had to represent a broad demographic group that reflected a significant proportion of visits to the ED (such as the elderly or children). We excluded studies that examined AE only among a subpopulation of the ED with a specific entrance complaint (such as respiratory diseases, cardiac complaints or trauma). We also excluded studies with patients receiving only a specific intervention (such as procedural sedation) and studies that reported only medication errors or adverse drug events. Finally, with respect to study setting, we included studies that provided both a numerator and a denominator for the proportion of ED patients who had an AE related to ED care. Studies conducted outside of the ED setting, including those that described AE in only hospitalized patients or in other ambulatory care settings, and did not provide specific data on the proportion of AE attributable to ED care, were excluded. Disagreements were resolved by consensus.

### Data Extraction

Data from each study was abstracted, using a standardized, piloted form, by one of two authors (AS or AP) with a third author (AW) verifying accuracy of extraction. Abstracted data included: study design, study definition of AE, participants, setting (ED type-i.e. urban, rural, academic, community, pediatric), data source, results (including number, type, preventability and severity of AE) and funding source. The primary outcome was the proportion of patients with AE related to ED care. Secondary outcomes included the preventability, the severity, and the types of AE (e.g., medication, procedure, diagnostic, sedation, or discharge related).

### Assessment of Methodological Quality

We rated methodological quality of included studies using the Newcastle-Ottawa Quality Assessment Scale (NOS) [18]. We felt that the NOS did not adequately assess important aspects of AE measurement and reporting, including methods for identifying AE, methods to establish causality between health care and outcome, and methods for establishing severity and preventability of AE. As a result we also utilized a tool adapted from a previous systematic review on adverse drug reactions, referred to hereafter as the Smyth adapted AE tool (Table 1) [19]. Two reviewers (AS, AP or AW) independently assessed the methodological quality of each study using the NOS and the Smyth adapted AE tool.

**Table 1:** Summary of Quality Assessments.

	Calder2010	Fordyce2003	Forster2007	Friedman2008	Hall2010	Hendrie2007a	Hendrie2007b	Henneman2005	Wolff2001	Wolff2002
**Newcastle-Ottawa Scale (NOS)**										
Selection^1^	****	***	***	****	****	****	****	***	****	****
Comparability^2^		*						**		
Outcome^3^	***		***		*			*	*	*
Total (out of maximum 9)	7	4	6	4	5	4	4	6	5	5
**Smyth Adapted AE Tool**										
**Study design**										
Clear study design	Yes	Yes	Yes	Yes	Unclear	Yes	Yes	Yes	Yes	Yes
**Methods to identify AE**										
Detailed methods to identify AE	Yes	Unclear	Yes	Yes	Unclear	Yes	Yes	Yes	Yes	Yes
Detailed data collection methods	Yes	Yes	Yes	Yes	Yes	Yes	Yes	Yes	Yes	Yes
Clear description of individuals identifying AE	Yes	Yes	Yes	Yes	Unclear	Yes	Yes	Yes	Yes	Yes
**Methods to identify causality**										
Clear description of process to establish causality	Yes	No	Yes	NR	NR	Yes	Yes	Yes	Yes	Yes
Standardized methods used to assess causality	Yes	No	Unclear	NR	NR	Yes	Yes	No	Yes	Yes
**Methods to determine preventability**										
Clear description of process to establish preventability	Yes	NR	Yes	Yes	NR	Yes	Yes	NR	No	NR
Standardized methods to assess preventability	NR	NR	NR	Unclear	NR	Yes	Yes	NR	No	NR
**Methods to determine severity**										
Clear description of process to establish severity	Unclear	NR	Yes	No	NR	Yes	Yes	No	Yes	Yes
Standardized methods to assess severity	Unclear	NR	Unclear	Unclear	NR	Yes	Yes	No	Yes	Yes
**Methods to determine type of AE**										
Clear description of process to establish type of AE	Yes	Yes	Yes	No	Yes	NR	Yes	NR	No	NR
Standardized methods to assess type of AE	Yes	Yes	Yes	Unclear	Unclear	NR	No	NR	Unclear	NR

1 Maximum of 4 stars for: representativeness of the exposed cohort; selection of the non-exposed cohort; ascertainment of exposure; and demonstration that outcome of interest was not present at start of study.

2 Maximum of 2 stars for: comparability of cohorts on the basis of the design or analysis.

3 Maximum of 3 stars for: assessment of outcome; was follow-up long enough for outcome to occur; and adequacy of follow-up of cohorts.

NR not reported.

### Analysis

We performed a qualitative analysis. The results reported for each study include setting, AE definition, proportion of patients with ≥1 AE related to ED care, method of determining causality, and AE preventability, severity and type (categorized by process of care). There was not enough data on children or the elderly to present a subset of results based on age.

## Results

### Study Selection

**Figure 1:**
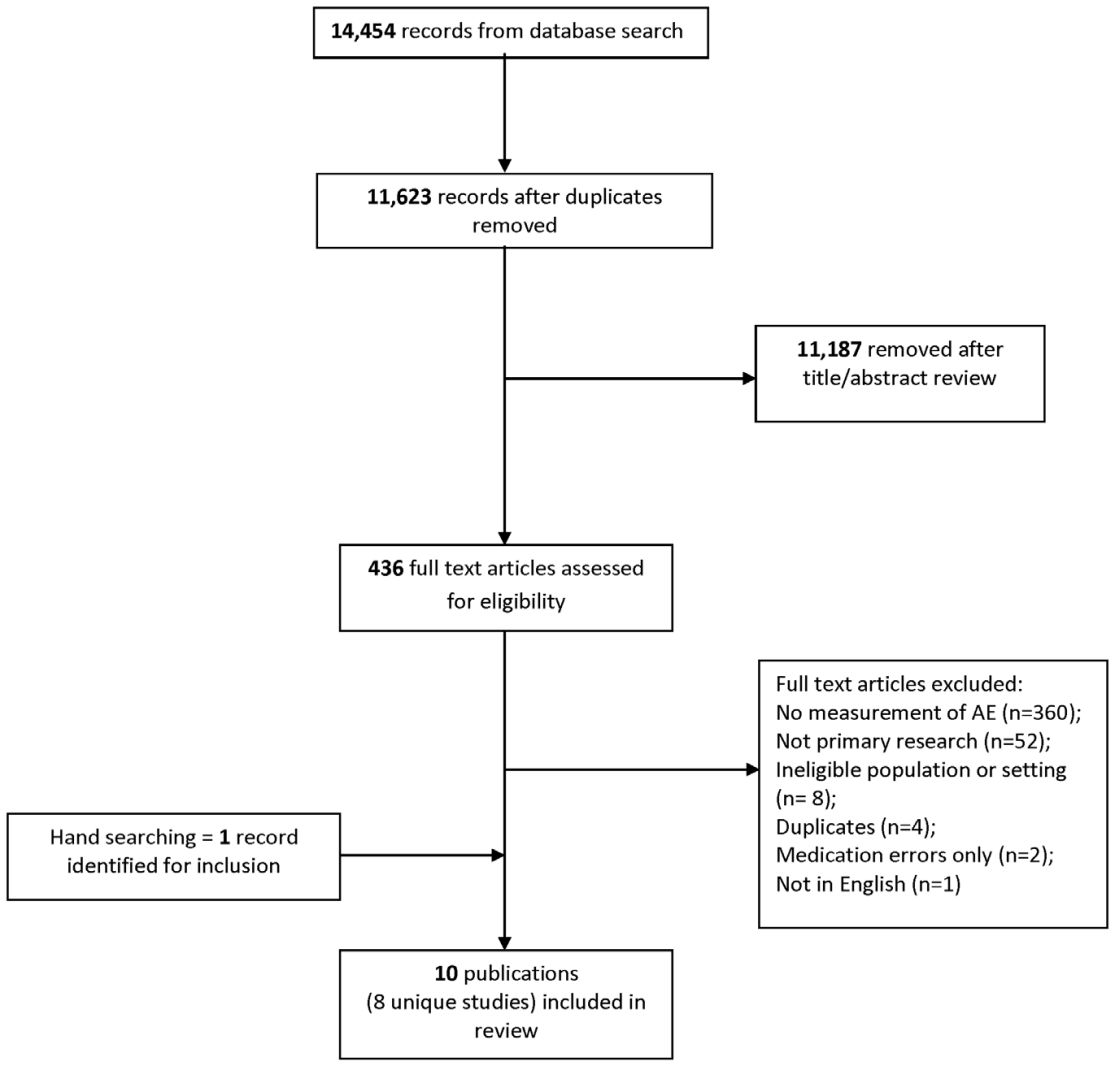
PRISMA Diagram.

The database search identified 14,454 potentially relevant citations with 11,623 citations after duplicate removal (Figure 1: PRISMA Diagram). Screening of titles and abstracts excluded 11,187 citations. We assessed 436 full text articles for eligibility and nine publications met the inclusion criteria. One article was identified in the hand search. A total of ten publications, representing eight studies (two studies reported results in two publications each [20], [21], [22], [23]) were included in the review.

### Study Characteristics

The included articles (Table 2 and Table S3) were prospective, observational studies: seven prospective cohort studies [9], [10], [20], [21], [24], [25], [26], [27] and one before-and-after interventional design [22], [23], [27]. Methodological quality was low to moderate with weaknesses in study group comparability, follow-up, and outcome ascertainment and reporting (Table 1). Particular weaknesses in outcome ascertainment and reporting included failure to clearly report methods used to determine AE causality, preventability and severity. Seven studies took place in urban, tertiary care academic hospitals in either Canada, the United States or Australia [9], [10], [21], [24], [25], [26], [27] and one study took place in a rural base hospital in Australia [22], [23]. A total of 38,702 patients were included in the eight studies, with a median of 1219 patients (range 201 [25] to 20,500 [22], [23]). Four studies included only adults [10], [24], [25], [26] and four enrolled adults and children [9], [20], [21], [22], [23], [27].

**Table 2:** Summary of Studies.

Study	Study type	Setting	Patients enrolled; Study	Adverse event (AE) definition	% patients ≥1 AE*	% preventable
Calder 2010	Prospective cohort study	2 tertiary care, academic urban hospitals; Canada	N = 518 (503 with follow- up); adults;	“…a flagged outcome associated with ED management.” Flagged outcomes included but were not limited to: new/worsening symptoms; an unscheduled visit to an ED or health professional; unscheduled hospital admissions or readmissions; unplanned transfer from another acute care hospital; death; litigation.	5%(25/503) attributable to ED care; 8.5%(43/503) overall (includes AE occurring after ED visit)	56%(24/43)
Hall 2010	Prospective observational study	Tertiary care, academic urban hospital; USA	N = 487 (482 with follow-up); adults;	“A non-ideal care event…any event in the patient’s care that the care giver judged to be less than ideal”; “Harm…any physical or psychological injury or damage to the health of a person, including both temporary and permanent injury.”	3%(13/482) visits with harm	Not reported
Friedman 2008	Prospective cohort study	Tertiary care, academic urban hospital; Canada	N = 201 (143 with follow-up); adults;	“Adverse event: unintended injury or complication caused by health care management rather than patient’s underlying disease.”	5%(10/201)	60%(6/10)
Forster 2007	Prospective cohort study	Tertiary care, academic urban hospital; Canada	N = 408 (399 with follow-up); adults;	“Adverse event = an injury due to treatment (an adverse outcome judged to be caused by medical management)”	6% (24/399)	71%(17/24)
Hendrie 2007a/b	Prospective observational study	Tertiary care, academic urban hospital; Australia	N = 5345patients (not consecutive; 3332 with follow-up); adults and children;	“Adverse event is: (i)unintended injury or complication, which (ii) resulted in death, disability, prolongation of the hospital stay, or prolongation of the natural history of the disease; and (iii) is caused by health care management rather than the patient’s disease.”	1.26%(42/3332) related to ED care; 3.12% (104/3332) (AE occurring both prior to and in ED)	55%(of AE occurring both prior to and in ED, no data provided for ED AE alone)
Henneman 2005	Prospective observational study	Tertiary care, academic urban hospital; USA	N = 9308; adults and children	“An adverse event…an injury or probable injury resulting from a medical intervention”.	0.16%(15/9308)	Not reported
Fordyce 2003	Prospective observational study	Tertiary care, academic urban hospital; USA	N = 1935 patients; adults and children	“Adverse event…an injury resulting from a medical intervention. Any incident causing pain, distress, or harm to a person was considered an adverse event”.	0.36%(7/1935)	Not reported
Wolff 2001/2002	Before and after interventional design	Rural base hospital; Australia	N = 20,500; adults and children	“…an untoward patient event which, under optimal conditions, is not a natural consequence of the patient’s disease or treatment”.	1.24%(250/20,050)	Preventable = 90(36%); potentially preventable = 156(62%)

* Refers to proportion of patients with AE related to ED care, unless otherwise specified.

### Primary Outcome

All eight studies reported the proportion of patients with ≥1 AE. The proportion of ED patients with AE related to ED care ranged from 0.16% (n = 9308) [27] to 6.0%(n = 399) [24] [10]. One study reported the proportion of patients with AE attributable to care provided in the ED (5.0%, n = 503) as well as the overall proportion of ED patients with AE events (8.5%, n = 503), which included events that occurred after the ED visit in both hospitalized and discharged patients [10]. A second study reported the proportion of patients with AE related to ED care (1.26%, n = 3332) as well as the total proportion of ED patients with AE (3.12%, n = 3332), which included events that occurred prior to the ED visit [20], [21]. Although one of the studies identified older age as a specific risk factor with the odds ratio (OR) for an AE highest for those age 61+ (1.66 (95% CI 1.26 to 2.19)) [22], and the one study (two publications) [22], [23] conducted in a community hospital reported a relatively low proportion of AE (1.24% n = 20,050)),there was not enough data to present results based on age or setting (ED type).

### Secondary Outcomes

Seven publications (representing five studies) [10], [20], [21], [22], [23], [24], [25] reported the preventability of AE which ranged from 36% (n = 250) [22], [23]to 71% (n = 24) [24]. Eight publications (six studies) [10], [20], [21], [22], [23], [24], [25], [27] provided data on the severity of AE. Most studies used a four to six point scale based on some combination of symptoms, prolongation of illness and response (e.g. ED visit, hospitalization); however, no validated, standard scale was used consistently between studies. Three studies reported the proportion of AE considered major 4% (n = 102) [27], 32% (n = 250) [22], [23] or significant 80% (n = 10) [25]. The proportion of patients with AE who required hospitalization was reported in two studies, 28% (n = 43) [10] and 42% (n = 24) [24]. Mortality related to AE was reported in five studies, 0.2% (n = 503) [10], 0.3%(n = 399) [24], 1.2% (n = 250) [22], [23], 0% (n = 9308) [27], 0.24% (for AE occurring prior to and in ED and 0.06% for AE occurring in the ED, n = 3332) [20], [21]. All eight studies reported on the types of AE. Management related events (e.g. pulmonary edema after excessively rapid infusion of normal saline) were the most common in 3 studies [9], [10], [24]; diagnosis related events (e.g. renal failure following delay in diagnosis of abdominal aortic aneurysm) were the most common in two studies (4 publications) [20], [21], [22], [23]; medication related events (e.g. anaphylaxis in patient with known codeine allergy prescribed codeine) were the most common in two studies [25], [27]; and procedural issues (e.g. difficulty in obtaining IV access) were the most common in the remaining study [26].

## Discussion

Previous systematic reviews have been conducted on the incidence and nature of in-hospital AE [28] and patient safety in the EMS/pre-hospital setting [29], however, to the best of our knowledge this is the first systematic review on AE related to ED care. The proportion of patients with AE related to ED care varied widely between studies. The proportion of patients with AE was higher in studies that utilized flagged outcomes and/or chart review [10], [20], [21], [22], [23], [24], [25] compared to those that used voluntary reporting or active solicitation of error reports [9], [26], [27]. Previous research has suggested that voluntary reporting systems detect less than 10% of AE [4], [17], [30]. The two highest quality studies in our review (based on both the NOS and the Smyth adapted AE tool) reported the highest proportions of ED patients with AE at 6% [24] and 8.5% (5% attributable to ED care) [10].

Previous research has identified particular risk factors for AE, including patient age and health care setting. For example, the pediatric population seen in the ED has been identified as particularly vulnerable to harm from medical errors [31], [32], [33] and research among hospitalized children has shown a high prevalence of safety events, comparable to that among hospitalized adults [34]. While four of the studies in this review enrolled both pediatric and adult patients, there were no pediatric specific studies and minimal age specific data reported. Patient safety studies in hospitalized patients have also identified older patients (≥65) as high risk for AE [4], [7]. Recent ED specific research has suggested that in elderly patients a prolonged ED length of stay is associated with an increased risk of an in-hospital AE [35]. However, little is known about the occurrence of AE in the ED among elderly patients. Despite the finding of older age as risk factor in one of the studies reviewed [22], there was insufficient information overall to present AE data based on age.

With respect to health care setting, even after adjustment for co-morbid conditions, previous work in hospitalized patients has demonstrated a higher number of AE in teaching hospitals compared to community hospitals for both adults and children [4], [14]. Of note, in the pediatric setting, although the overall proportion of AE was higher in teaching hospitals, the proportion of AE attributable to the ED was higher in community hospitals (15.9% (n = 69) vs 5.7% (n = 210)) [14]. Although one study (two publications) [22], [23] in our review was conducted in a community hospital, the substantial variation in methods and reporting between studies prevented any comparisons based on study setting.

The results of this review suggest that a substantial proportion of AE related to ED care may be preventable. The preventability of AE occurring in the ED (36% to 71%) is at least comparable to that reported in previous studies of hospitalized patients (35% to 51%) [4], [6], [7], [8]. Previous work in hospitalized patients demonstrated that the largest proportion of AE were operative [4], [14], [28], [36] and medication related [4], [8]. In the ED setting, management, diagnosis and medication related events were common. One of the studies in this review also identified differences in the preventability and types of AE among discharged and admitted patients [10]. A greater proportion of AE were preventable among the discharged population (71.4%; n = 15) than among the admitted population (40.9%; n = 9). Among discharged patients, management issues (47.6%; n = 10), diagnostic issues (33.3%; n = 7), and unsafe dispositions decisions (19%; n = 4) were the most common causes of AE. In comparison, admitted patients more commonly suffered from procedural complications (50%; n = 11), management issues (36.4%; n = 8) and medication adverse effects (27.2%; n = 6). These findings suggest that the types of AE that occur in the ED may be different than in hospitalized patients, and different between patients who are discharged from the ED and those who are admitted. These results support the need for setting specific research and intervention.

### Limitations

The strengths of this review include an up-to -date, comprehensive search strategy, clearly defined eligibility criteria and standardized data extraction. The main weakness of the review is the dearth of high quality studies in this area. Study quality was low to moderate with significant variability and weaknesses in outcome assessment and reporting, which may have accounted in part for the wide variability in the prevalence and preventability of AE. In addition, there was some variability in the definition of AE among the included studies which limits the generalizability of the results. A particular weakness of many of the studies was the limited time frame of AE measurement, with no follow-up outside of the index ED visit [9], [20], [21], [22], [23], [26], [27]. The studies with a longer timeframe (7–14 days post ED visit) [10], [24], [25] reported a higher proportion of AE, which suggests that the studies without follow-up may have substantially underestimated AE related to ED care. A final weakness of this review is the lack of a standardized, validated tool for critically appraising the quality of observational studies measuring health care related harm. We attempted to address this by completing both the NOS scale [18] for observational studies as well as an adaptation of a tool [19] specifically developed to assess the quality of studies looking at medication related harm (adverse drug reactions). Although the systematic review process was methodologically rigorous, the existing research in this area is poor, with many of the studies being relatively small and having multiple flaws.

## Conclusions

The methodological flaws of the individual studies and the variability in definition of AE among the included studies precludes broad, generalized conclusions with respect to the prevalence, preventability and severity of AE related to ED care. The lack of large, high quality studies on AE in the high risk ED setting is, however, in itself an important finding and highlights the need for further research in this area. In particular, further research is required to better understand specific risk factors for AE in the ED, including patient age and ED setting. Future studies in this area should include an adequate duration of follow-up to capture AE attributable to ED care that occur after discharge, and should use rigorous and standardized outcome reporting, especially with respect to AE causality, preventability and severity.

## Supporting Information

Table S1 **Medline Search Strategy.**
(DOC)

Table S2 **Journals and Conference Proceedings.**
(DOC)

Table S3 **Detailed Summary of Included Studies.**
(DOC)

Protocol S1 **Protocol.**
(DOCX)

Checklist S1 **PRISMA Checklist.**
(DOC)
