# Supplementary material for: Adverse Events Related to Emergency Department Care: A Systematic Review
Source: PLoS One. 2013 Sep 12;8(9):e74214. doi: 10.1371/journal.pone.0074214 (PMC3772011; doi:10.1371/journal.pone.0074214)
Supplement: Table S1 — Medline Search Strategy. (DOC) [file pone.0074214.s001.doc]

**Table S1: Medline* Search Strategy**

| 1. Emergency Medicine/ |
| --- |
| 2. emergency treatment/ |
| 3. Emergencies/ |
| 4. exp Emergency Medical Services/ |
| 5. Emergency Medical Technicians/ |
| 6. exp ambulances/ |
| 7. Critical Care/ |
| 8. (EDs or ED or ERs or ER or EMS).tw. |
| 9. (emergenc* adj2 (medic* or health or hospital*) adj2 service*).tw. |
| 10. (emergenc* adj (department* or room* or ward* or unit* or care of hospital* or patient* or physician* or doctor* or treatment*)).tw. |
| 11. (emergenc* adj (medic* or service*)).tw. |
| 12. (emergenc* adj2 (technician* or practitioner* or dispatch*)).tw. |
| 13. (prehospital* or pre-hospital*).tw. |
| 14. (trauma adj (center* or centre*)).tw. |
| 15. out of hospital*.tw. |
| 16. paramedic*.tw. |
| 17. ambulance*.tw. |
| 18. (transport* adj2 (medic* or patient* or air)).tw. |
| 19. or/1-18 |
| 20. exp Accident Prevention/ |
| 21. exp Medical Errors/ |
| 22. exp Health Services Misuse/ |
| 23. Inappropriate Prescribing/ |
| 24. Malpractice/ |
| 25. Iatrogenic Disease/ |
| 26. Infectious Disease Transmission, Professional-to-Patient/ |
| 27. exp Equipment Failure/ |
| 28. Adverse Drug Reaction Reporting Systems/ |
| 29. Drug Toxicity/ |
| 30. safe* manage*.tw. |
| 31. patient* safe*.tw. |
| 32. (Medica* adj4 error*).tw. |
| 33. (error* adj3 (health care or healthcare)).tw. |
| 34. (adverse adj (event* or outcome* or react*)).tw. |
| 35. (sentinel adj2 event*).tw. |
| 36. (critical* adj3 (incident* or outcome*)).tw. |
| 37. (unanticipated adj2 outcome*).tw. |
| 38. (diagnos* adj2 (error* or lack*)).tw. |
| 39. (underdiagnos* or under diagnos* or misdiagnos* or mis* diagnos*).tw. |
| 40. (error* adj3 (nurs* or physician* or patient care or surg* or human*)).tw. |
| 41. (safe* adj3 (cultur* or climate*)).tw. |
| 42. (equipment adj3 fail*).tw. |
| 43. or/20-42 |
| 44. and/19,43 |
| 45. randomized controlled trial.pt. |
| 46. controlled clinical trial.pt. |
| 47. randomized.ab. |
| 48. exp Clinical Trials as Topic/ |
| 49. randomly.ab. |
| 50. trial.ab. |
| 51. or/45-50 |
| 52. exp animals/ not humans/ |
| 53. 51 not 52 |
| 54. Epidemiologic studies/ |
| 55. exp case control studies/ |
| 56. exp cohort studies/ |
| 57. Case control.tw. |
| 58. (cohort adj (study or studies)).tw. |
| 59. Cohort analy*.tw. |
| 60. (follow up adj (study or studies)).tw. |
| 61. (observational adj (study or studies)).tw. |
| 62. longitudinal.tw. |
| 63. retrospective.tw. |
| 64 cross sectional.tw. |
| 65. Cross-sectional studies/ |
| 66. or/54-65 |
| 67. meta analysis.mp,pt. |
| 68. review.pt. |
| 69. search*.tw. |
| 70. or/67-69 |
| 71. or/53,66,70 |
| 72. and/44,71 |
| 73. limit 72 to humans |
| 74. limit 73 to english language |
| 75. remove duplicates from 74 |

*The search strategy was translated as appropriate for the other databases
