# Supplementary material for: Adverse Events Related to Emergency Department Care: A Systematic Review
Source: PLoS One. 2013 Sep 12;8(9):e74214. doi: 10.1371/journal.pone.0074214 (PMC3772011; doi:10.1371/journal.pone.0074214)
Supplement: Table S2 — Journals and Conference Proceedings. (DOC) [file pone.0074214.s002.doc]

**Table S2: Journals and Conference Proceedings**

| **Journals** | **Conference Proceedings** |
| --- | --- |
| Academic Emergency Medicine | American Academy of Pediatrics (AAP) |
| Annals of Emergency Medicine | American College of Emergency Physicians (ACEP) |
| BMJ Quality and Safety | Canadian Association of Emergency Physicians (CAEP) |
| Canadian Journal of Emergency Medicine | Canadian Pediatric Society (CPS) |
| Canadian Medical Association Journal | Pediatric Academic Societies (PAS) |
| Journal of Patient Safety | Society for Academic Emergency Medicine (SAEM) |
| Journal of Pediatrics |  |
| Pediatrics |  |
