# Supplementary material for: Adverse Events Related to Emergency Department Care: A Systematic Review
Source: PLoS One. 2013 Sep 12;8(9):e74214. doi: 10.1371/journal.pone.0074214 (PMC3772011; doi:10.1371/journal.pone.0074214)
Supplement: Table S3 — Detailed Summary of Included Studies. (DOC) [file pone.0074214.s003.doc]

**Table S3: Detailed Summary of Included S**tudies

| **Study/**  **Study type/**  **Setting** | **Patients enrolled/**  **Study timeframe/**  **Window of scrutiny** | **Adverse event (AE) definition** | 1. **Method of record selection/patient recruitment** 2. **Method of review** | **Causation** | **% patients ≥ 1 AE** | **% preventable** | **Severity** | **AEs* categorized by process of care** |
| --- | --- | --- | --- | --- | --- | --- | --- | --- |
| **Calder 2010**  Prospective cohort study  2 tertiary care, academic urban hospitals;  Canada | N=518 (503 with follow- up); adults;  Discharged and admitted patients (5 month study period);  2 weeks post discharge from ED or within 2 weeks of admission | “…a flagged outcome associated with ED management.”  Flagged outcome:  Discharged patients: new/worsening symptoms; an unscheduled visit to an ED or health professional; unscheduled hospital admissions; death  Admitted patients:  Unplanned transfer from another acute care hospital, to acute monitoring area and/or neuro-observation area and/or ICU; unplanned transfer or return to operating room; organ damage/removal during surgery; hospital complications; development of new neurologic deficits; hospital-acquired infection; accident or injury; adverse drug reaction; dissatisfaction with care; litigation; unplanned readmission to hospital | 1. Random assignment of RN to enrolment shifts (24 hrs a day, 7 days a week), consecutive patients registered in resuscitation or observation areas of ED; 2. Adverse outcomes flagged and summarized by reviewer and telephone interview (2 weeks after discharge) post-discharge ED visit or hospitalization. Two specialist physicians independently rated outcome summaries to determine if AE occurred. | 6-pt Likert scale ≥4, two independent reviewers deemed AE | 5%(25/503) attributable to ED care;  8.5%(43/503) overall (includes AE occurring after ED visit) | 56%( 24/43);  6-pt Likert scale,  2 reviewers | 6-pt severity scale (N=43);  Abnormality on lab testing= 6 (14%);  ≤1 day symptoms= 42%(18/43);  > 1 day symptoms= 37%(16/43);  Nonpermanent disability= 1(2%)  Permanent disability= 1(2%)  Death= 1(2%)  Response (N=43):  Required medical/surgical intervention= 20(47%);  Admission to hospital= 12(28%);  No treatment (symptoms) = 10(23%);  ED visit= 4(9%);  Visit to MD office= 2 (5%) | **(N=43):**  Management= 18(42%)  Procedural= 13(30%)  Diagnostic= 10(23%)  Medication= 9(21%)  Infection= 6(14%)  Disposition= 4(9%)  Fall= 3(7%)  Follow-up= 2(5%) |
| **Hall 2010**  Prospective observational study  Tertiary care, academic urban hospital; USA | N=487 (482 with follow-up); adults;  Admitted and discharged patients (15 week study period);  ED visit only | “A non-ideal care event…any event in the patient’s care that the care giver judged to be less than ideal”  “Harm…any physical or psychological injury or damage to the health of a person, including both temporary and permanent injury.” | 1. Patient care givers (attending, resident, RN) contacted within 1 hour of patient’s discharge 2. RAs interviewed all available care givers for sampled patient visits, collected basic demographic data and visit characteristics | Not reported | 3%(13/482)  visits with harm | Not reported | Majority of AEs related to multiple IV attempts | **Non-ideal care events (N=14) with known harm:**  Procedural issue= 8(57%)  Management= 3(21%)  Medication= 1(7%)  Radiology= 1(7%)  Laboratory testing= 1(7%) |
| **Friedman 2008**  Prospective cohort study  Tertiary care, academic urban hospital; Canada | N=201 (143 with follow-up); adults;  Discharged patients (3 month study period);  Up to 7 days post- discharge | “Adverse event: unintended injury or complication caused by health care management rather than patient’s underlying disease.” | 1. ED patients recruited for participation in standardized interview within 24hrs of discharge and follow-up interview 3-7 days post-discharge 2. Interviewer reviewed medical chart and hospital patient and visitor incident database; ED physician categorized incidents using pre-defined criteria for adverse event, near miss and medical error | Not reported | 5%(10/201) | 60%(6/10) | 5-pt scale;  Significant 80%(8/10);  Moderate 20%(2/10) | **Majority of AEs (N=10) were related to pain management:**  Medication= 6(60%)  Procedure= 2(20%)  Clinical services= 2(20%) |
| **Forster 2007**  Prospective cohort study  Tertiary care, academic urban hospital; Canada | N=408 (399 with follow-up); adults;  Discharged patients (10 week study period);  2 weeks post-discharge | Adverse event=an injury due to treatment (an adverse outcome judged to be caused by medical management)  “Adverse outcome…experienced new or worsening symptoms; visited an ED; were readmitted to hospital; or died.” | 1. Investigator (present in ED for 8-10 hour shifts M-F 8am-6pm) approached patients likely to be discharged home 2. Adverse outcome flagged and summarized by study author using chart review; telephone interview (2 weeks post- discharge). Two specialist physicians independently rated outcome summaries to determine if AE occurred | 6-pt Likert scale: ≥4 deemed AE | 6% (24/399) | 71%(17/24) | 4-pt scale;  Hospitalized=42%(10/24);  Symptoms=38%(9/24);  ED visit=21%(5/24) | **Preventable AE (N=17):**  Management=7(29%)  Diagnostic=6(25%)  Inadequate follow-up=3(13%)  Unsafe disposition=1(4%) |
| **Hendrie 2007a/b**  Prospective observational study  Tertiary care, academic urban hospital; Australia | N=5345patients (not consecutive; 3332 with follow-up); adults and children;  Admitted and discharged patients (2 month study period);  ED visit only | “Adverse event is: (i)unintended injury or complication, which (ii) resulted in death, disability, prolongation of the hospital stay, or prolongation of the natural history of the disease; and (iii) is caused by health care management rather than the patient’s disease.” | 1. Convenience sample during study week; 5 identified by “word of mouth” 2. Review by 2 reviewers to determine AE (1 identified; 1 reviewed identified cases) | 6-pt Likert scale; ≥4 deemed AE | 1.26%  (42/3332 events occurred in ED)  3.12%(104/3332 events occurred prior to ED and in ED) | 55% (of AE occurring both prior to and in ED, no data provided for ED AE alone) | 6-pt scale;  Illness prolonged <1 month=48% (AE occurring both prior to and in ED)  Mortality ( from AE occurring both prior to and in ED)= 0.24%(8/3332)  Mortality (from AE occurring in ED= 0.06%(2/3332) | **Diagnostic problems and errors of omission were significantly associated with ED events:**  Diagnostic issues=76%(32/42) |
| **Henneman 2005**  Prospective observational study  Tertiary care, academic urban hospital; USA | N=9308; adults and children;  Admitted and discharged patients (31 day study period);  ED visit only | “An adverse event…an injury or probable injury resulting from a medical intervention”.  “Error…the failure of a planned action to be completed as intended or the use of an incorrect plan to achieve an aim”.  “Near miss…an error that had been recovered before it affected the patient”. | 1. ED staff voluntarily completed safety report; 2 authors categorized events as having adverse impact on patient 2. Review by 2 authors into minor/potentially adverse events or serious adverse events | Adverse effect determined by ED staff reporting | 0.16% (15/9308) | Not reported for AE  75% of errors (152/203) were preventable | Increased length of stay= 56%  Minor/potentially AE=11%(11/102);  Major AE= 4%(4/102) | **Errors (203 errors; 15 AEs):**  Medication related=33%  Miscommunication=30%  Test-related=24% |
| **Fordyce 2003**  Prospective observational study  Tertiary care, academic urban hospital;  USA | N=1935 patients; adults and children;  7 day study period;  ED visit only | “Adverse event…an injury resulting from a medical intervention. Any incident causing pain, distress, or harm to a person was considered an adverse event”.  “Error…the failure of a planned action to be completed as intended or the use of an incorrect plan to achieve an aim”. | 1. Active solicitation of error reports from all care providers (nurses, MDs, other) every 3-4 hours – staff determined AE? 2. 2 independent reviewers determined categorization of error to “area of care”; consensus panel for disagreements | Determined by ED staff making report; no scale reported | 0.36% (7/1935) | Not reported | Not reported | **AEs (N=7):**  Clinical management=4(57%)  Diagnostic studies=2(29%)  Pharmacotherapy=1(14%)  **Errors (N=346):**  Diagnostic studies=77(22%)  Administrative procedures=56(16%)  Pharmacotherapy=54(16%)  Documentation=45(13%)  Communication=43(12%)  Environmental=39(11%)  Clinical management=10(3%)  Team functioning=7(2%)  Triage=7(2%)  History and exam=4(1%)  Other=4(1%) |
| **Wolff 2001/2002**  Before and after interventional design  Rural base hospital; Australia | N=20,500; adults and children;  2 year study period;  ED visit only | “…an untoward patient event which, under optimal conditions, is not a natural consequence of the patient’s disease or treatment”. | 1. Database screening for 1 of 5 criteria: death, unplanned representation to department within 48hrs for same condition, length of stay>6hrs, transfer 2. Positive criteria records screened by hospital clinical risk manager for AE and subsequent review by director of medical services | 6-pt Likert scale; ≥4 | 1.24% (250/20,050) | Preventable= 90(36%);  Potentially preventable= 156(62%) | 6-pt scale:  Major AE=81(32%)  Minor AE=169(68%) | **Errors associated with AEs (N=250); some AEs involved >1 error:**  Avoidable delays in diagnosis= 49(20%)  Inadequate functioning of hospital service= 49(20%)  Inadequate reporting/communication= 40(16%)  Technical error= 26(10%)  Practicing outside area of expertise= 25(10%)  Avoidable delay in treatment= 20(8%) |

*Unless otherwise noted, the processes of care categories are for adverse events (some studies reported errors or ‘non-ideal care events’); numerators are provided if they were reported in the studies
