# Supplementary material for: Adverse Events Related to Emergency Department Care: A Systematic Review
Source: PLoS One. 2013 Sep 12;8(9):e74214. doi: 10.1371/journal.pone.0074214 (PMC3772011; doi:10.1371/journal.pone.0074214)
Supplement: Protocol S1 — Protocol. (DOCX) [file pone.0074214.s004.docx]

**Adverse Events Related to Emergency Department Care: A Systematic Review**

PROTOCOL

| Title | **Adverse Events Related to Emergency Department Care: A Systematic Review** |
| --- | --- |
| Authors  Search Librarian  Article Retrieval  Study Coordination | Antonia S Stang, Aireen S Wingert , Lisa Hartling, Amy C Plint  Andrea Milne  Teodora Radisic  Denise Thomson |
| Corresponding Author | Antonia S Stang |
| Background | 1. There is a high prevalence of adverse events (AE , defined as unintended harms resulting from the care and services provided to the patient) among hospitalized patients. 2. The ED is considered high risk for patient safety events. 3. Previous research has identified population and setting specific differences in the prevalence and types of AE. 4. Patient safety work conducted on hospitalized patients may not be generalizable to the emergency department (ED) setting. 5. There is a significant knowledge gap on the existing evidence on patient safety specific to the ED. |
| Objectives | To review the current evidence on AE occurring in the ED setting. |
| Types of studies | Randomized clinical trials (RCTs) and observational studies |
| Types of participants | Included:  All patients presenting to the ED  Studies whose participants reflect the overall ED population or a broad demographic group that reflect a significant proportion of visits to the ED (such as the elderly or children).  Studies that address both measurement of adverse outcomes and measurement of the association of these outcomes with the ED care provided.  Excluded:  Outpatients, those from pre-hospital, ambulatory or community settings.  Studies that examine AE only among a subpopulation of the ED with a specific entrance complaint or receiving a specific intervention – such as adverse drug events, procedural sedation or cardiac intervention. |
| Types of outcome measures | Primary outcome   1. Prevalence of AEs (number or proportion of AEs) related to ED care.   Secondary outcomes   1. Types of AEs (medication, procedure, diagnostic, sedation, or discharge related) 2. Preventability of AE 3. Severity of AE |
| Search methods for identification of studies | Databases:  Medline (1985 to present),  Cochrane Library (2005 to November 2011),  International Pharmaceutical Abstracts (1985 to December 2011),  EMBASE (1985 to present),  PubMed (last 180 days),  CINAHL (1985 to present),  Web of Knowledge (1985 to present)   - MeSH terms such as adverse outcome or patient safety, and emergency - Screening by two reviewers, disagreements to be resolved by a third reviewer |
| Data collection and analysis | Abstraction by one author with independent data verification by a second author using a standardized form (piloted)  Quantitative   1. Data:  - Study design - Study definition of AE - Sample size - Participant characteristics - Setting - Data source  1. Results: proportion of patient visits with AE related to ED care, 2. Subgroup analysis by age (pediatric (≤18), adult (>18 and < 65) and elderly (≥65) or as defined by study authors)   Qualitative   1. Description of the types of AE, preventability of AEs, and severity of AEs.   Methodological quality assessment:   - Cochrane’s Risk of Bias tool (controlled studies) - Newcastle-Ottawa Quality Assessment Scale (case-control and retrospective cohort studies) - Smyth adapted AE tool for all studies |
| Contributions of authors | Literature search: AM  Screening: AP, AS  Article retrieval: TR  Data abstraction form: AW, AP, AS  Data extraction: AP, AS, AW  Risk of Bias/quality assessment: AP, AS, AW  Methodological advice: LH  Data analysis: AP, AS, AW,LH  Results write-up: AS  Final analysis and write-up: AS  Manuscript completion: AS, AP, AW,LH |
| Conflicts of interest | None |
| Sources of support | Funding provided by the CIHR Team Grant in Pediatric Emergency Medicine |
